# Epstein-Barr virus infection and vitamin D deficiency are both “causal” for multiple sclerosis (MS) - could the common denominator be their effects on hepcidin levels?

**DOI:** 10.1007/s11011-025-01678-8

**Published:** 2025-10-04

**Authors:** Susan J. van Rensburg, Ronald van Toorn, Mariaan Jaftha, Merlisa C. Kemp, Penelope Engel-Hills, Maritha J. Kotze

**Affiliations:** 1 Division of Chemical Pathology, Department of Pathology, Faculty of Medicine and Health Sciences, Stellenbosch University, Cape Town, South Africa; 2 Department of Pediatrics and Child Health, Faculty of Medicine and Health Sciences, Stellenbosch University, Cape Town, South Africa; 3 Department of Medical Imaging and Therapeutic Sciences, Faculty of Health and Wellness Sciences, Cape Peninsula University of Technology, Cape Town, South Africa; 4 Cape University Body Imaging Centre, Faculty of Health Sciences, University of Cape Town, Cape Town, South Africa; 5 Medical Imaging, Department of Health and Care Professions, Faculty of Health and Life Sciences, University of Exeter, Exeter, UK; 6 Faculty of Health and Wellness Sciences, Cape Peninsula University of Technology, Cape Town, South Africa

**Keywords:** Multiple sclerosis, EBV reactivation, Vitamin D, Hepcidin, Iron, Demyelination

## Abstract

Multiple sclerosis (MS) is a neurological disorder characterized by damage to the myelin sheaths surrounding axons in the central nervous system, causing decreased axonal signal transmission and disability in people with MS. Epstein-Barr virus (EBV) infection and vitamin D deficiency have been put forward as causal factors for the development of MS, but their effects have not been conclusively linked to the disruption of myelin maintenance. Interestingly, both EBV infection and vitamin D deficiency increase the levels of hepcidin, an acute-phase peptide hormone that inhibits iron absorption. The current understanding of iron dysregulation in MS is that iron accumulates in deep gray matter brain structures which leads to disability progression. However, recent studies have revealed that the apparent iron influx may be an artefact of disease-related brain atrophy, and that iron is in contrast *depleted* in the deep gray matter in MS, which could cause iron deficiency in oligodendrocytes (the cells producing myelin), leading to their demise due to a mitochondrial energy deficit, with consequent demyelination. EBV infection, vitamin D deficiency and iron deficiency may converge as causal risk factors for MS. Dismantling the current understanding that iron excess underpins MS would improve testing and optimization of iron parameters and vitamin D as part of clinical management of MS. This review additionally explores the risk factors for lytic reactivation of EBV which is hypothesized to drive MS disease activity. Conversely, ensuring that EBV remains in a latent state by ameliorating these risk factors may prevent MS exacerbations and disease worsening.

## Introduction

Multiple sclerosis (MS) is an inflammatory neurological disorder affecting the central nervous system (CNS), characterized by damage to the myelin sheaths surrounding the neuronal axons, which disrupts signal transmission within the brain and from the brain to peripheral organs, leading to disability. However, clinical symptoms vary significantly among people with MS (pwMS) (DeLuca et al. 2007; van Rensburg et al. 2021a). While some individuals experience mild, occasional symptoms, others suffer from severe incapacitation (DeLuca et al. 2007). Notably, this disparity in disability outcome appears unrelated to treatment with disease-modifying therapies (DMTs) (Tremlett et al. 2012; Jaftha et al. 2024) or genetic factors on their own, despite the identification of over 50 genomic regions associated with MS susceptibility (Disanto et al. 2012). For example, the HLA-DRB1*15:01 gene variant is associated with higher risk of MS diagnosis, but not with disability progression (Barcellos et al. 2006; Van der Walt et al. 2011). The diagnosis and progression of MS are influenced by several environmental risk factors, including deficiencies as well as aggravators (Munger and Ascherio 2013; Jaftha et al. 2024). This complexity rules out a single cause for MS. As the primary goal of MS treatments is to reduce disability, the potential risk factors have been extensively studied with the aid of neurological tests such as the Expanded disability status scale (EDSS), allowing for objective assessment of disability in pwMS (Kurtzke 1983).

Two environmental risk factors considered causal for MS are vitamin D deficiency and Epstein-Barr virus (EBV) infection; however, their respective mechanisms of action have not been established (Munger and Ascherio 2013; Giovannoni 2024). Low vitamin D status and high anti-EBV nuclear antigen 1 (EBNA-1) IgG antibody titers are independent risk factors for MS, although it has been shown that vitamin D supplementation could improve the immune response against EBV (Disanto et al. 2013). Three Mendelian randomization studies (useing genetic information to ascertain a causal relationship between a risk factor and a disease) performed from 2015 to 2017, confirmed the relationship between vitamin D and MS. The first study found that each genetically determined one-standard-deviation decrease in log-transformed 25(OH)D level conferred a 2.0-fold increase in the odds of MS, while elevated serum vitamin D reduced the odds of MS by approximately 50% (Mokry et al. 2015). A second study, involving two separate populations in California and Sweden, showed that increased levels of 25(OH)D were associated with a decreased risk of MS in both populations, giving a combined odds ratio (OR) of 0.85 (*p* = 0.003, 95% confidence interval (CI) 0.76–0.94) (Rhead et al. 2016). The third study found that the negative relationship between vitamin D and MS also held true for pediatric-onset MS: vitamin D-associated genetic risk scores (GRS) that are associated with increased levels of 25(OH)D in serum, decreased the odds of pediatric-onset MS, OR 0.72 (*p* = 0.02, 95% CI 0.55–0.94) after controlling for sex, genetic ancestry, HLA-DRB1*15:01, and non-human leukocyte antigen MS risk variants. Notably, this study also demonstrated a significant association between body mass index (BMI), GRS and earlier onset of pediatric MS, with an OR of 1.17 (*p* = 0.01, 95% CI 1.05–1.30) (Gianfrancesco et al. 2017).

## Epstein-Barr virus (EBV)

A second important risk factor for MS, EBV, was first recognized by Warner and Carp (1981), followed by the formulation of a hypothesis that EBV was involved in MS etiology (Warner and Carp 1988). EBV was established as the leading cause of MS in a large cohort study of more than 10 million young adults in the US military, where the risk of MS increased 32-fold after EBV infection (Bjornevik et al. 2022). This study suggested that EBV infection was necessary (but insufficient) for the development of MS (Giovannoni 2024). EBV is a DNA virus belonging to the Human B lymphotropic herpesvirus family. It has a global infection rate exceeding 90%. After the acute phase, EBV persists as a latent infection, primarily in B cells (Sausen et al. 2021). In immunocompetent individuals, this latent infection is not typically associated with clinical symptoms (Kerr 2019). However, immunodeficiency can facilitate virus reactivation, which is linked to various diseases, including cancer, infections and immune-related disorders such as MS, systemic lupus erythromatosis (SLE), rheumatoid arthritis, inflammatory bowel disease, juvenile idiopathic arthritis and celiac disease (Harley et al. 2018; Sausen et al. 2021). In pwMS, antibodies to both latent and lytic EBV proteins are detected, suggesting that EBV drives MS disease activity through latent-lytic infection cycles (Giovannoni 2024). Antibodies to various EBV antigens, such as viral capsid antigen (VCA), EBNA1, and early antigen (EA, also known as BMRF1) have been measured in the blood (Hon et al. 2012; Duque et al. 2023), as well as in some MS brain lesions (Orr and Steinman 2025) and in some oligoclonal bands (Castellazzi et al. 2014). Anti-VCA IgM appears early in the infection while anti-VCA IgG may persist for life. During the latent phase, EBV may not exacerbate MS symptoms; for example, Gieß et al. (2017) found no association between EBNA-1 nor VCA IgG antibodies in serum, EBV DNA load in saliva, and radiological or clinical disease activity in patients with clinically isolated syndrome (CIS) or early relapsing-remitting MS (RRMS). Additionally, they found no link between these factors and the conversion of CIS to clinically definite MS. Hon et al. (2012) confirmed the presence of anti-EBV-VCA IgG in over 99% of both pwMS and controls, while anti-EBV-VCA IgM positivity was significantly higher in pwMS (41.9%) compared to controls (23.3%; *p* = 0.046). Notably however, anti-EBV IgM, indicating a primary immune response, was associated with relapses, suggesting that active viral particles may have contributed to these relapse episodes (Hon et al. 2012).

## Iron dysregulation as a third potential risk factor for MS

Iron dysregulation has been proposed as a potential risk factor for MS, specifically oxidative iron overload. Numerous studies have reported iron deposition and increased iron concentrations in the brains of pwMS, which appear to correlate with age, disease duration and disability (Gemmati et al. 2012). This concept of elevated brain iron in neurodegenerative diseases, including MS, Parkinson’s disease and Alzheimer’s disease, has influenced the perceptions of researchers over many years, with iron chelation put forward as a possible treatment option (Bsteh et al. 2019; Ayton et al. 2025). However, decreased iron in certain brain areas of pwMS that were related to brain atrophy, have also been observed (Schweser et al. 2021). This has led to a recent understanding of magnetic resonance imaging (MRI) that there is a distinction between iron *concentration* as measured by R2* and iron *content* of the brain in MS. Importantly, the prevailing theory that increased magnetic susceptibility in MS indicates iron accumulation in deep gray matter may be flawed. Disease-related atrophy may be the underlying cause of these observations (Schweser et al. 2021). This new understanding suggests that, contrary to current beliefs, iron is being *depleted* from deep gray matter in pwMS, rather than accumulation (Schweser et al. 2021). Another study emphasizes this conclusion by its title: “Increased mean R2* in the deep gray matter of multiple sclerosis patients: Have we been measuring atrophy?” (Hernández-Torres et al. 2019). A study measuring iron changes over five years in RRMS, found thalamic atrophy and decreased iron in regions of the caudate, putamen, and thalamus (Elkady et al. 2019). Notably, a recent randomized controlled trial (RCT) of deferiprone, an iron chelator, in patients with amyloid-confirmed early Alzheimer’s disease (AD), yielded surprising results. Decreasing hippocampal iron concentration was accompanied by worsened cognitive decline and increased volume loss in frontal areas, as evidenced by declining performance in executive function tests. The authors concluded that lowering iron levels with deferiprone was detrimental to cognitive function and brain health in patients with AD (Ayton et al. 2025).

CNS atrophy, resulting in volume loss, is significantly associated with disability progression in MS (Ghione et al. 2018; Tsagkas et al. 2021). Loss of brain tissue in pwMS is also associated with the death of myelin-producing oligodendrocytes (Barnett and Prineas 2004; Jaftha et al. 2024). Oligodendrocytes have an essential requirement for iron which is related to energy production in their mitochondria (Connor and Menzies 1996; van Rensburg and Van Toorn 2010; van Rensburg et al. 2012). Gerber and Connor (1989) furthermore demonstrated that oligodendrocytes are the main iron-containing cells in the brain. Interestingly, a recent Mendelian randomization analysis study on iron dysregulation in MS, suggested that iron deficiency may also be a causal factor for MS. This study revealed a potential causal relationship between MS and transferrin (Tf), with an OR of 1.22 (*p* < 0.001, 95% CI: 1.10–1.36) and Tf saturation, with an OR of 0.75 (*p* = 0.02, 95% CI: 0.75–0.98) (Tang et al. 2024). Elevated serum Tf levels are associated with decreased serum iron, while increased Tf saturation indicates iron repletion. Iron is delivered to the brain by the iron transport molecule Tf, and the amount of iron bound to Tf is reported as % Tf saturation. Clinically, iron status is assessed by measuring key parameters, including hemoglobin, serum iron, Tf, Tf saturation and ferritin (the iron storage protein) (Auerbach et al. 2025;  Tang et al. 2024).

## Interaction of EBV and vitamin D with the iron regulatory system

**Fig. 1:**
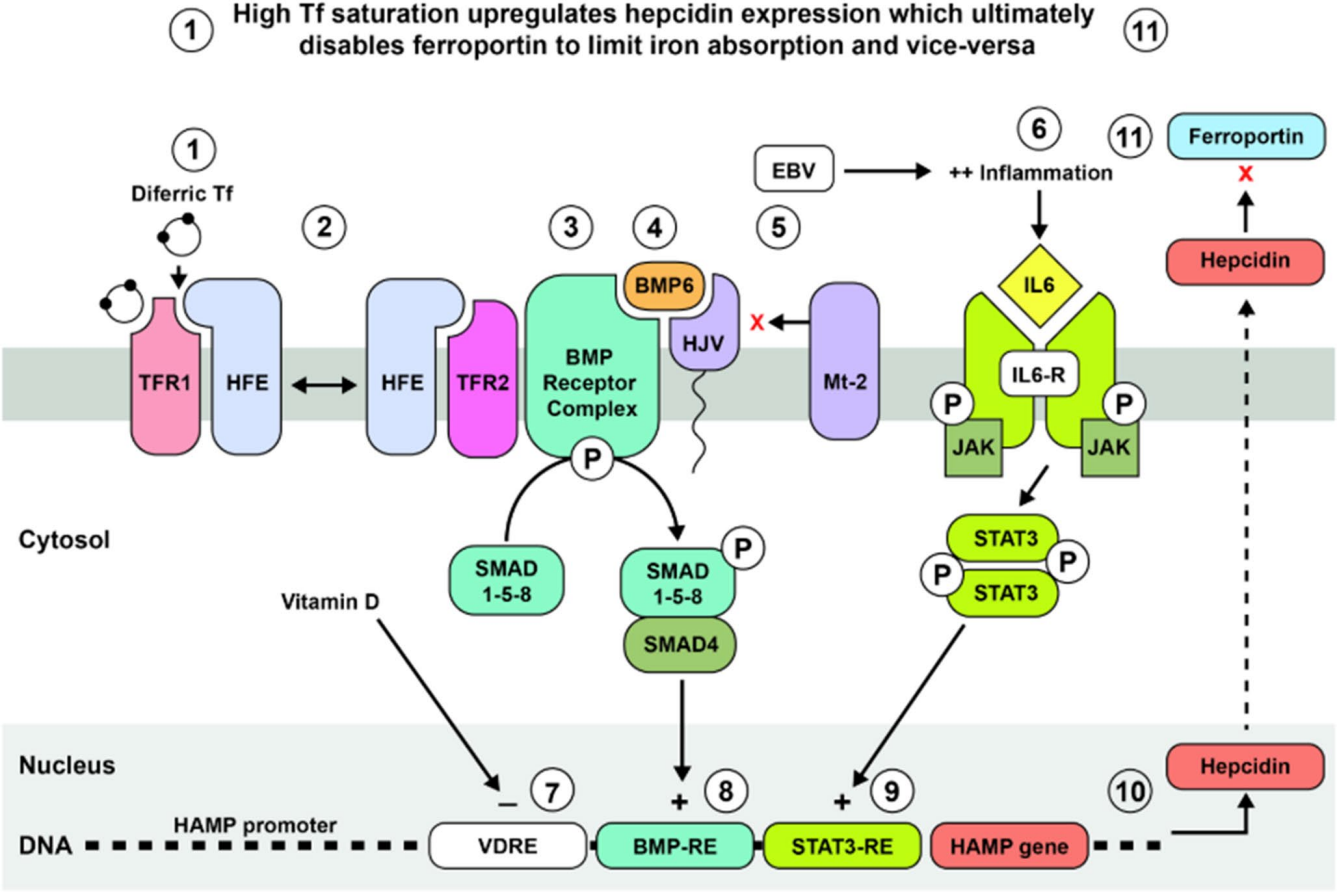
Schematic representation of mechanisms involved in iron regulation, showing examples of molecules involved, using the hepatocyte as a model

The interactions of vitamin D and EBV with the iron regulatory system are described in Box 1 and depicted in Fig. 1. Iron regulation is a highly complex process, involving multiple components for which models have been developed and refined (Nemeth and Ganz 2023). Maintaining optimal iron levels in the body and brain is crucial, as sufficient iron is essential for life, while excessive iron can cause oxidation and cell damage. Central to this regulatory process is the ability of cells such as hepatocytes, to “sense” the iron status inside cells and in the blood (Tf saturation) (Lawen and Lane 2013), which then controls iron absorption by regulating the synthesis of the peptide hormone hepcidin, through transcriptional regulation of the *HAMP* (hepcidin antimicrobial peptide) gene (Lawen and Lane 2013). Hepcidin synthesis relies on a complex multistep signaling process that is influenced by interactions between external factors such as EBV and vitamin D with protein molecules located on the hepatocyte membrane (Fig. 1). Two primary signaling pathways in hepatocytes regulate hepcidin production: the BMP-SMAD135/SMAD4 signaling pathway, which senses blood iron levels though the binding of Tf to the Tf receptors Tf1 and Tf2, and the IL-1β/IL-6 inflammatory pathway. These pathways modulate hepcidin production in response to changes in iron levels and inflammatory signals to maintain iron homeostasis in the body (Pippard 2017).Hepcidin regulates iron levels by inhibiting the function of ferroportin, the protein responsible for transporting iron out of duodenal enterocytes and loading it onto Tf molecules in the blood. A deficiency in ferroportin also traps iron within macrophages that recycle iron from ageing red blood cells, thereby reducing the export of iron to Tf from these macrophages. In essence, hepcidin synthesis acts as a master switch to control serum iron levels (Lawen and Lane 2013). Elevated hepcidin levels and decreased ferroportin therefore reduce the amount of iron bound to Tf which in turn would decrease the amount of iron available to cells such as oligodendrocytes.

Figure 1 is a graphic representation of the metabolic process described in Box 1.

Figure 1: The iron regulatory system responds to the concentration of iron in the blood: High Tf saturation (1) upregulates hepcidin expression through numerous on/off switches which ultimately (11) disables ferroportin to limit iron absorption. Low Tf saturation has the opposite effect: hepcidin expression is decreased and iron absorption is increased. Examples of on/off switches are shown in the diagram, e.g. phosphorylation/dephosphorylation is a separately regulated on/off signaling mechanism. (1) Tf can exist in apo-, mono-, and diferric forms. When diferric Tf binds to TFR1, it displaces HFE, which then binds to TFR2 (2) (HFE toggle switch). (3) This enables the BMP Receptor complex to phosphorylate SMAD 1-5-8 in the presence of BMP6 and HJV. (4) The expression and activity of BMP6 is separately regulated by hepatic iron levels, while (5) the activity of HJV is negatively regulated by Mt-2 (TMPRSS6), a protease that disables HJV. (6) EBV activates the inflammatory signalling pathway. Inflammation upregulates hepcidin expression through binding of IL6 to IL6-R which causes the phosphorylation and activation of the JAK/STAT3 signalling pathway. Hepcidin expression is further regulated by the binding of various molecules to response elements (REs) of the HAMP gene promoter, such as (7) vitamin D which binds to VDREs to suppress hepcidin expression (Bacchetta et al. 2014). Vitamin D also downregulates inflammation by decreasing IL6 (Zughaier et al. 2014). Hepcidin expression is upregulated by (8) phosphorylated SMAD 1-5-8 and SMAD4 which together bind to the BMP-RE and (9) phosphorylated STAT3 which binds to the STAT3-RE. (10) All these reactions together determine the amount of hepcidin which circulates via the blood to (11) block ferroportin from allowing iron uptake and -release into plasma, thereby lowering systemic iron levels (Lawen and Lane 2013; Pippard 2017; Nemeth and Ganz 2023).

Abbreviations: Tf: Transferrin; HAMP: hepcidin antimicrobial peptide; TFR1: transferrin receptor 1; TFR2: transferrin receptor 2; HFE: Hereditary hemochromatosis protein; BMP: bone morphogenic protein; SMAD: small mother against decapentaplegic; Mt-2: matriptase-2; TMPRSS6: transmembrane protease serine 6; IL6-R: Interleukin-6 (IL6) receptor; JAK/STAT: Janus kinase (JAK) and signal transducer and activator of transcription (STAT).

## The clinical effect of EBV on hepcidin

Despite the evidence linking EBV to MS, it is not known how EBV causes MS, even after nearly 60 years of continuous investigation (Yu and Robertson 2023). Proposed mechanisms have included molecular mimicry, immune dysregulation, and activation of MS-associated human endogenous retroviruses (Giovannoni 2024; Madeira et al. 2016).

A novel finding which may shed light on this subject, is the report by Duque et al. (2023) of a significant positive association between EBV infection and elevated serum hepcidin levels. In a cohort of children from boarding schools in Mexico City, 145 EBV-infected children exhibited higher levels of acute phase proteins, including hepcidin, α−1 glycoprotein (AGP) and C-Reactive protein (CRP) levels compared to uninfected children (Duque et al. 2023). Regarding EBV, 91.7% of the children were positive for one or more of the antibodies against EBV antigens and all of them were positive for VCA-IgG, indicating past infection or that the children were EBV carriers. Stronger associations were observed between hepcidin and antibodies against lytic markers VCA and EA than with antibodies against the latent protein EBNA1. In an extended study, a similar association was found between EBV and hepcidin upregulation in adults, in the database of gastric cancer of the transcriptomic and genomic (TCGA) consortium (*N* = 250). The data in the TCGA database allowed analysis of the two pathways controlling hepcidin expression, BMP − SMAD and IL-1β/IL-6. Only the IL-1β/IL-6-dependent inflammatory pathway and their signalling intermediaries JAK2 and STAT3 were significantly associated with EBV infection (Duque et al. 2023).

There is a paucity of studies in the literature reporting hepcidin levels in pwMS. Ellidag et al. (2014) found no difference between either hepcidin or iron parameters in pwMS compared to controls, while Bsteh et al. (2019) reported increased hepcidin concentrations in pwMS compared to controls: 26.9 ng/ml (CI 22.8–30.9) vs. 17.3 ng/ml (CI 12.8–23.4); however, after adjusting for age and sex the difference was not significant. In a normal reference population, serum hepcidin levels varied according to age in women, being higher in postmenopausal than premenopausal women, but were not associated with age in men (Galesloot et al. 2011). In addition, hepcidin concentrations have been shown to follow a circadian rhythm induced by melatonin (Park et al. 2022). Zierfuss et al. (2023) commented that despite varying results, most studies of pwMS have found lower levels of serum iron but higher amounts of ferritin in the circulation, suggesting that elevated hepcidin may play a role, along with genetic and environmental factors. Future studies should be done to establish whether hepcidin levels would be specifically associated with disease activity and inflammation in pwMS during lytic reactivation of EBV, as discussed below.

Inflammation in MS (“smoldering MS” or the “real” MS) (Giovannoni et al. 2022) may influence hepcidin levels and iron parameters due to elevated IL-6 (Fig. 1). Frei et al. (1991) measured IL-6 in cerebrospinal fluid (CSF) and plasma of pwMS, muscular tension headache (TH) and acute meningo-encephalitis (AM). PwMS had higher levels of IL-6 in plasma compared to patients with TH and AM (Frei et al. 1991). Elevated CSF levels of IL-6 were associated with new inflammatory brain lesions, unlike in pwMS with low or absent IL-6 concentrations, who had a better disease course (Stampanoni Bassi et al. 2019).

In other neurological diseases also associated with EBV infection, significant associations with hepcidin and iron concentrations have been demonstrated, especially during exacerbations. Serum hepcidin is increased significantly together with decreased iron parameters in SLE (Kunireddy et al. 2018). Serum hepcidin levels are associated with disease activity in rheumatoid arthritis patients and may play a role in the pathobiology of chronic disease anaemia associated with rheumatoid arthritis (Demirag et al. 2009). Serum hepcidin is increased in children with inflammatory bowel disease and it is responsible for iron malabsorption (Soltanieh et al. 2024), and in juvenile idiopathic arthritis hepcidin levels were significantly higher in anaemic and active disease patients (Troiani et al. 2025).

## Latitude and MS - effects of vitamin D and EBV

Epidemiological data have consistently shown a link between vitamin D deficiency and MS. Apart from diet or supplementation, vitamin D is acquired through sunlight absorbed by the skin. While the global number of people with MS has increased from 2.3 million in 2013 to 2.9 million in 2023, the distribution of MS cases varies significantly across the world (MS International Federation. Atlas of MS 2023). According to the Atlas, the highest prevalence of MS (expressed as the number of cases per 100 000) is in the Northern countries such as Canada and Scandinavia. South America has fewer cases, and the lowest prevalence is in Sub-Saharan Africa near the equator. (Table 1) Interestingly, EBV seropositivity increases with distance from the equator (Disanto et al. 2013) while EBV exhibits an inverse association with vitamin D levels in both pwMS and controls.

**Table 1:** Number of people with MS and Prevalence per 100,000 people in different countries (MS International Federation. Atlas of MS)

Country	Number of people with MS	Prevalence per 100,000 people
Canada	90,000	290
USA	913,925	288
Norway	13,765	255
United Kingdom	133,780	199
Argentina	17,017	38
Brazil	40,000	19
South Africa	4,685	8
Namibia	49	4
Zambia	20	0.1
Tanzania	6	0.01

Munger et al. (2006) validated a link between serum vitamin D levels and MS risk in a prospective nested case-control study among more than 7 million US military personnel. They found 257 cases of MS which were matched to controls. The risk of MS increased significantly with lower vitamin D blood levels especially for those under the age of 20 years. The level of serum vitamin D also modifies disease activity in pwMS (Sintzel et al. 2018). Vitamin D has many pleiotropic actions on the immune system resulting in immunomodulation through promoting regulatory subsets of T cells and B cells rather than effector T and B cells, modulating the activity of innate immune cells such as monocytes, macrophages and dendritic cells, and reducing immune cell trafficking at the blood-brain barrier (BBB) (Galoppin et al. 2022). Vitamin D exerts additional activity within the CNS to reduce the activation of microglia and astrocytes (Galoppin et al. 2022). Vitamin D also modulates the immune/inflammation system by inhibiting the proliferation of proinflammatory cells and by regulating the production of inflammatory cytokines, both of which are associated with the pathogenesis of inflammatory diseases. Lower vitamin D status is related to the increased risk of acute infections and chronic inflammatory diseases such as MS, chronic kidney disease, non-alcoholic fatty liver disease, atherosclerosis-related cardiovascular disease, asthma, inflammatory bowel disease, and others (Yin and Agrawal 2014).

### Vitamin D suppresses hepcidin, decreases inflammation and increases iron concentrations

Several studies have found that vitamin D suppresses hepcidin concentrations (Smith et al. 2017). Bacchetta et al. (2014) showed that vitamin D decreases hepcidin by binding directly to vitamin D response elements (VDREs) in the promotor region of the *HAMP* gene (Fig. 1). In cultured monocytes and hepatocytes, vitamin D’s metabolites, 25-hydroxyvitamin D and the hormonally active form of vitamin D, 1,25-dihydroxyvitamin D, directly inhibited hepcidin mRNA expression by binding to identified consensus VDREs within a 1071-bp *HAMP* proximal promoter DNA sequence (Bacchetta et al. 2014). Treatment with vitamin D increased expression of ferroportin in hepatocytes and monocytes. In the same study, healthy volunteers receiving a single oral dose of 100,000 IU of vitamin D2, had increased circulating levels of vitamin D and 34% decreased hepcidin levels at 24 h after supplementation (Bacchetta et al. 2014). Another double-blind placebo controlled trial randomized 28 healthy adults to receive a one-time oral dose of 250,000 IU of vitamin D3 or placebo, and found that vitamin D significantly decreased circulating hepcidin concentrations by 73% from baseline (*p* < 0.005) (Smith et al. 2017). Zughaier et al. (2014) also reported the results of a double blind RCT of oral vitamin D3, 50,000 IU weekly for 12 weeks, followed by 50,000 IU every other week for 40 weeks or matching placebo for one year. Increased serum vitamin D was significantly inversely associated with decreased hepcidin concentrations (*r* = 0.38, *P* = 0.02) and positively associated with hemoglobin and iron concentrations in vitro and in vivo. Since elevated hepcidin inhibits iron uptake from the gut and sequesters iron in the reticuloendothelial system, this results in anaemia of inflammation associated with the release of the inflammatory cytokines IL-6 and IL-1β from monocytic cells. Treatment with vitamin D reduces the concentrations of these two hepcidin stimulatory cytokines in chronic kidney disease (CKD) (Zughaier et al. 2014). Vitamin D also induces the expression of the antibacterial protein cathelicidin in macrophages (Bacchetta et al. 2014; Zughaier et al. 2014).

In cultured monocytic cells stimulated by lipopolysaccaride (LPS), 1,25(OH)2D3 resulted in a dose-dependent decrease in the release of IL-6 and IL-1β, together with decreased hepcidin and increased ferroportin expression (Zughaier et al. 2014). IL-6 mediates hypoferremia of inflammation by inducing the synthesis of hepcidin (Nemeth et al. 2004).

## The hidden dangers of iron deficiency

The hidden health risks of iron deficiency anemia (IDA) were highlighted by Chang et al. (2020) who explored the relationship between the diagnosis of IDA and autoimmune diseases to ascertain which of these occurred first. The researchers analyzed data from Taiwan’s National Health Insurance research database, which included a random sample of 1 million people. They identified 22,440 patients with IDA and 89,528 without IDA to investigate the temporal association between IDA and autoimmune disease diagnoses. The results showed that patients with IDA had a significantly higher risk of developing autoimmune diseases, with an adjusted hazard ratio (HR) of 2.37 (95% CI 1.92–2.92) compared to the non-IDA group. This increased risk was particularly evident within 2 years *after* an IDA diagnosis, especially in females and patients with certain comorbidities. The diagnosis of IDA was based on the following criteria: Hb concentration less than 12 g/dL for females and less than 13 g/dL for males; mean corpuscular volume (MCV) of less than 80 fL and a ferritin concentration of less than 15–30 ng/mL (Chang et al. 2020). The autoimmune diseases diagnosed according to ICD-9-CM codes included rheumatoid arthritis, SLE, Sjögren’s syndrome, systemic sclerosis, as well as polymyositis and dermatomyositis. Celiac disease was excluded from the study due to its low prevalence in Taiwan (Chang et al. 2020). Similarly, MS has a low prevalence of 5 per 100,000 people in Taiwan (MS International Federation. Atlas of MS 2023). However, a Canadian study, where the prevalence of MS is 290 per 100,000 (Table 1), found that anemia forms part of the prodrome of MS (symptoms experienced during the five years preceding the first demyelinating event) with an adjusted OR of 1.53 (Yusuf et al. 2021).

Iron deficiency can have detrimental effects on brain health. For example, iron chelation with deferiprone in patients with AD worsened brain deterioration and cognition (Ayton et al. 2025). Additionally, serum hepcidin levels are increased in individuals at risk for AD (Chatterjee et al. 2020) and in patients with Parkinson’s disease compared to controls (Kwiatek-Majkusiak et al. 2020). Long-COVID-19 has also been associated with iron dysregulation, increased hepcidin and anaemia of inflammation (Lechuga et al. 2023; Hanson et al. 2024; Suriawinata and Mehta 2023). Furthermore, studies have found associations between IL-6 with EBV levels in COVID-19 patients (Lehner et al. 2020). Moran-Lev et al. (2018) found an association between hepcidin, vitamin D and anaemia in children with acute infections. These findings highlight the complex relationship between iron deficiency, hepcidin dysregulation, and various autoimmune and neurodegenerative diseases.

## Treatments for MS - are they safe and effective?

The term “causal” when describing risk factors for MS may be misleading, as it implies that a single risk factor may cause MS and that eliminating this risk will cure the disease. Vitamin D supplementation, for example, has already been evaluated in several RCTs. However, the results have been disappointing, leading Hawkes et al. (2019) to lament that instead of caviar the researchers received a dog’s dinner (Hawkes et al. 2019). A recent RCT of a very high dose of 100,000 IU oral cholecalciferol or placebo every 2 weeks for 24 months, showed a 14% risk reduction in disease activity without the anticipated side-effects; the median time to progression was also significantly longer than in the placebo group, but the treatment did not prevent relapses (Thouvenot et al. 2025), suggesting that vitamin D supplementation alone may not address all the underlying causes of MS, highlighting the complexity of MS and the need for a more comprehensive treatment approach.

The established causal link between EBV and MS has sparked interest in more invasive treatments (Kerr 2019; Giovannoni 2024). Given the universal presence of EBV infection and its potential to cause immune dysregulation, researchers have proposed vaccination to prevent primary EBV infection or boosting the immune response to EBV using mRNA vaccines (Giovannoni 2024). Since memory B cells harbor latent EBV, targeting B cells has been considered a valid treatment for MS (Rød et al. 2023). Most licensed DMTs have been observed to target B cells, either by reducing their numbers or depleting them (Giovannoni 2024). Additionally, drugs with anti-EBV effects, such as highly active antiretrovirals (HAART), including tenofovir, famciclovir or other antiviral agents have been considered for treating MS (Labella et al. 2021; Maruszak et al. 2011). However, as EBV is not the sole risk factor for MS and the mechanisms by which EBV contributes to MS are not fully understood, the results of such clinical trials may also be inconclusive. Interestingly, antiretroviral therapy has been found to alleviate anemia in HIV patients, and HAART medications such as tenofovir have been shown to lower hepcidin levels in patients with HIV (Oluboyo et al. 2022). In addition, other herpesviruses have also been identified as potential risk factors for MS, such as Varicella-zoster virus (VZV) and Human herpes virus 6 (HHV-6) (Khalesi et al. 2023).

If EBV affects the hepcidin pathway and decreases iron levels (Duque et al. 2023), eliminating the virus through vaccination may not impact MS directly, because pwMS can experience iron deficiency through various mechanisms. Our research group has identified non-anemic iron deficiency in children with MS due to iron-lowering genetic variants using whole exome sequencing (WES) (Van Toorn et al. 2010; van Rensburg et al. 2019). We have also documented MS diagnosis and relapses triggered by blood loss through multiple childbirths (Johannes et al. 2023) and blood donation (van Rensburg et al. 2012, 2016). We have additionally observed flare-ups of MS symptoms during menstruation and prevention thereof by increasing iron supplementation before menstruation, as well as low iron intake due to veganism (unpublished results). Notably, our published case series demonstrated remission in pwMS when iron deficiency and other risk factors were addressed (van Rensburg et al. 2021b; Johannes et al. 2023; Jaftha et al. 2024).

**Fig. 2:**
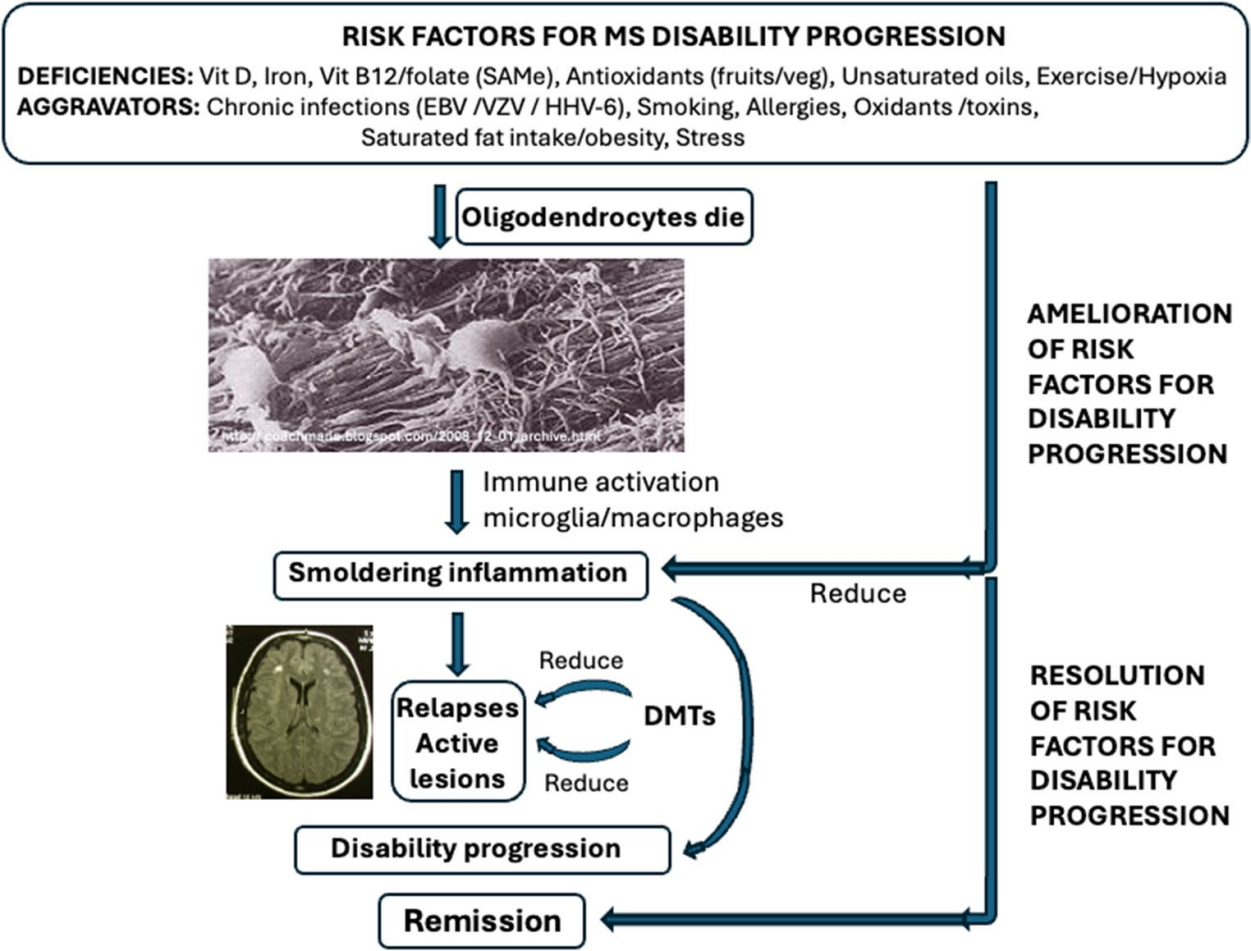
Oligodendrocyte cell death and consequent immune activation to clear away dead cells and debris (Barnett and Prineas 2004; Prineas and Parratt 2021) may underlie smouldering inflammation, demyelination, and disability progression in pwMS. While DMTs reduce relapses and slow active white matter lesion formation, they do not resolve smouldering inflammation (Giovannoni et al. 2022). However, resolution of the risk factors for oligodendrocyte death and disability progression may effectively result in remission (Jaftha et al. 2024). Oligodendrocyte image adapted from http://coachmarla.blogspot.com/2008_12_01_archive.html DMTs: disease modifying treatments

Current and proposed invasive medical treatments for MS can have significant drawbacks, including adverse events that some patients may find intolerable. Hohlfeld (2010), giving the 2009 lecture at the European Committee for Treatment and Research in Multiple Sclerosis (ECTRIMS) meeting, highlighted five challenges for MS research that had to be resolved: (1) ensuring that the price of MS drugs complied with medical ethics; (2) optimization of the risk/benefit ratio for MS treatments, since increasing efficacy was found to be accompanied by increased risk for adverse events; (3) bridging the gap between MS and experimental autoimmune encephalomyelitis; (4) promoting neuroprotection and repair; and (5) ‘tailoring’ MS therapy to the individual patient (Hohlfeld 2010). None of these 5 challenges have been resolved. According to the World Health Organization VigiBase^®^, long-term administration of MS DMTs may increase cancer risk, with ORs ranging from 1.15 to 1.74 (Dolladille et al. 2021). B cells have a complex role in cancer progression, with different phenotypes promoting or inhibiting tumour growth (Largeot et al. 2019). B cells also play a crucial role in wound healing and immune function (Sîrbulescu et al. 2017), increasing the risk of serious infections if immune function is altered by DMTs (Langer-Gould et al. 2023). Furthermore, the efficacy of DMTs at preventing disease progression decreases with age: a meta-analysis of 38 RCTs found that the efficacy of DMTs to prevent disability fell to zero beyond approximately age 53 years (Weideman et al. 2017), since DMTs do not resolve smouldering inflammation despite reducing relapses and slowing active white matter lesion formation (Giovannoni et al. 2022). In their neuropathological studies of early MS lesions, Barnett and Prineas (2004) showed that the death of oligodendrocytes underlies the disease process in MS; subsequently, Prineas and Parratt (2012) observed apoptosis of oligodendrocytes immunoreactive for caspase 3 with subsequent phagocytosis of the apoptotic oligodendrocytes (Prineas and Parratt 2021). Therefore, resolving the risk factors for oligodendrocyte cell death (deficiencies and aggravators) (Jaftha et al. 2024) , may contribute to remission in MS, rather than a single medical intervention (Fig. 2).

## A new research strategy

The goal of MS research is to investigate methods to prevent disease worsening and disability, and to promote sustained remission without damaging side-effects (Hohlfeld 2010). There is an unmet need to develop new strategies to prevent smouldering inflammation (Giovannoni et al. 2022). If EBV indeed drives MS disease activity through the latent-lytic cycle (Giovannoni 2024; Hon et al. 2012), a more effective strategy would arguably be to prevent reactivation of the virus to prevent disability progression. Given that 95% of the human population and virtually 100% of pwMS have to live with EBV, considering factors that prevent EBV activation may be important for many different disorders as individuals may remain asymptomatic during EBV latency (Kerr 2019; Sausen et al. 2021). A connection between EBV and the regulation of apoptosis was observed by Fitzsimmons and Kelly (2017). Evidence is provided in Box 2 that all the risk factors for MS related to oligodendrocyte cell death described previously (Jaftha et al. 2024) may result in the reactivation of EBV and conversely, that alleviation of these risk factors may prevent EBV reactivation. For example, iron chelation rapidly induces the synthesis of lytic EBV antigens (Kraus et al. 2017) as well as apoptosis (Jiang et al. 2002), whereas holo-Tf blocks apoptosis (Fassl et al. 2003). Furthermore, impairment of vitamin B12 metabolism (Orozco-Barrios et al. 2009) and folate deficiency (Zhang et al. 2009) also initiate apoptosis. Notably, zinc was shown to be essential for the oligomerization of EBNA1 which keeps EBV in the latent state (Jiang et al. 2019). Interventions for risk reduction to ensure adequate nutrient levels, minimize toxic exposures, and promote optimal cellular function were associated with significantly smaller cerebral white matter lesion volumes and sustained remission in pwMS in a published case series (Jaftha et al. 2024).

## Conclusion

The discovery that the biochemical mechanism underlying the disease-related risk for MS of both EBV infection and vitamin D deficiency involves hepcidin, the master iron uptake inhibitor, may inform the development of future treatments of disorders such as MS. Since it is impossible to guess a patient’s iron status from clinical presentation alone, the only way to determine it is by requesting comprehensive iron parameter blood tests to monitor iron levels and track improvements. However, convincing clinicians that testing iron parameters would be clinically valid for MS may depend on the time it would take to dismantle the current understanding that brain iron accumulation underlies disability in pwMS. Furthermore, EBV may not exacerbate disease when it is in its dormant phase; therefore, it may be reasonable to investigate ways to prevent EBV reactivation as a major goal of disease management. Optimization of iron, vitamin D and vitamin B12/folate metabolism is crucial to achieve optimal myelin synthesis and maintenance, along with adequate intake of nutraceuticals present in fruits and vegetables, as well as regular exercise to prevent hypoxia. Additionally, avoiding serial EBV reactivators such as smoking is essential. Future research should establish whether adopting a comprehensive program to promote a healthy lifestyle could lead to resolution of smouldering inflammation and less invasive treatments with fewer side-effects. Ultimately, the goal of MS research is to prevent disease progression and disability. All avenues should be explored to achieve this objective.

**Box 1:** The iron regulatory pathway

**The Multi-step Process involved in Iron Homeostasis** Tf plays a crucial role in iron homeostasis. When the body experiences iron deficiency, hepatocytes increase Tf production, making more transport molecules available to bind iron entering the bloodstream from the gut. Each Tf molecule can bind one or two iron atoms (apo-, monoferric- and diferric/holo Tf). Iron is internalized into cells by the binding of Tf to TFR1 by which iron is carried into hepatocytes that "sense" that there is sufficient iron in the blood (Lawen and Lane 2013). When holo Tf binds to TFR1, HFE detaches from TFR1 and binds to TFR2 which then together form a complex with BMP6 and HJV (Figure 1). This complex increases the transcription of the HAMP gene through the BMP-SMAD signaling pathway, to increase the production of hepcidin (Nemeth and Ganz 2023). Hepcidin then prevents further iron release from the enterocytes, as well as iron release from immune cells, through blocking ferroportin function. Conversely, when iron levels decrease, Tf saturation decreases, TFR1 is available to bind to HFE which dissociates from the BMP complex, the transcription of hepcidin is decreased, ferroportin becomes functional and allows iron to flow into the blood (Figure 1). Tf saturation is therefore one of the pivotal control elements in iron regulation. We have previously found associations of Tf saturation with disease parameters in MS: Tf saturation was associated significantly with the fractional anisotropy of white matter tracts, a marker for myelin functionality (p < 0.01) and inversely associated with disease duration (p= 0.02). Disability assessed with the EDSS was also inversely associated with Tf saturation in 76 non-smoking females (p = 0.05) (Herbert et al. 2018), with optimal values > 20 % (Herbert et al. 2018; van Rensburg et al. 2021b). **Genetic variations in the iron regulation pathway in MS** At any given time, iron intake through the diet or genetic variations affecting any of the proteins involved in iron regulation may complicate the regulatory process by causing iron overload or iron deficiency. Genetic variations have been found to influence hepcidin synthesis: the HFE C282Y and H63D single nucleotide variants (SNVs) cause inability of the HFE protein to bind to HJV, leading to a decrease in hepcidin synthesis and iron overload (Figure 1). The same occurs with genetic variations in TF, HJV, TFR1 and TFR2 (Figure 1). Genetic polymorphisms in the protease matriptase-2 (TMPRSS6) cause an inability to cleave HJV (i.e. by removing the negative regulator, the “brake”); consequently, hepcidin synthesis is unopposed with resulting iron deficiency. The variants with opposing effects on iron uptake can also work against each other to cancel their clinical impact. The iron excess hypothesis for MS is not reflected by the presence of SNVs in the HFE gene: a meta-analysis has shown that the HFE polymorphisms C282Y and H63D are not associated with susceptibility to MS (Starčević Čizmarević et al. 2022). An earlier study by Kotze et al. (2006) discovered that when comparing genetic data of pwMS with pathology tests (liver function tests, radioisotope studies of the liver and with iron parameters), pwMS who were homozygous for HFE C282Y did not show signs of pathological iron deposition in the liver, and that some of the pwMS who had heterozygous HFE variants unexpectedly had low serum iron parameters. This led us to hypothesize that another variant was modulating the anticipated HFE effect. Moremi et al. (2018) provided evidence that that the iron-lowering variant most likely was TMPRSS6 c.2207C>T A736V (rs855791), as the combined genotype effect of HFE and TMPRSS6 was associated with significantly reduced ferritin levels and earlier age of onset in pwMS (Moremi et al. 2018). MS is also associated significantly (p<0.01) with allelic variants of the NRAMP1 promoter polymorphism (Kotze et al. 2001). NRAMP1 is expressed in phagocytic cells and participates in the recycling of iron acquired from phagocytosed senescent erythrocytes by releasing iron from recycled erythrocytes into the cytoplasm of monocytes (Soe-Lin et al. 2009). In addition, vitamin D increases the expression of NRAMP1 (Zughaier et al. 2014).
**The role of Infection/Inflammation in hepcidin synthesis and iron regulation**Hepcidin levels are elevated in response to inflammation or infections, whether bacterial or viral (Rodriguez et al. 2014). As an antimicrobial peptide, hepcidin plays a crucial role in the body’s defense against pathogens. When immune cells detect invading microorganisms, they release inflammatory cytokines such as IL-6. These cytokines activate the IL-1β/IL-6 signaling pathway in hepatocytes, leading to increased hepcidin synthesis (Figure 1). As a result, iron uptake and release from immune cells is prevented, which would likely withhold iron from invading pathogens to prevent their multiplication during infection (Rodriguez et al. 2014). However, this mechanism may also have negative consequences, depriving other cells such as oligodendrocytes of essential iron for functions such as myelin synthesis (Jaftha et al. 2024). Infections are environmental triggers of disease exacerbation in MS (Steelman 2015).

**Box 2:** Evidence that the risk factors for MS (deficiencies and aggravators) (Jaftha et al. 2024) cause reactivation of EBV and that alleviation of these risk factors may prevent EBV reactivation

Deficiencies	Aggravators
Vit D	Chronic infections
Iron	Smoking
Vit B12/folate	Allergies
Antioxidants (fruits/veg)	Oxidants/toxins
Unsaturated oils	Saturated fat intake/obesity
Exercise/Hypoxia	Stress
**Vitamin D** deficiency is associated with EBV reactivation and vitamin D controls EBV by modulating the T cell-mediated response to EBV (Hedström et al. 2023). Vitamin D supplementation prevents EBV reactivation (Najafipoor et al. 2015, Disanto et al. 2013). **Iron deficiency** rapidly induces the synthesis of lytic EBV antigens (Kraus et al. 2017). Incubation with deferoxamine, an iron chelator, promoted lytic EBV antigens in both epithelial and lymphocytic EBV+ cells within 24 hours through stabilization of hypoxia-inducible factor alpha (HIF-α) (Kraus et al. 2017). While iron supplementation has not yet been tested as a means to prevent EBV reactivation, Chao et al. (2012) found that the risk factors for reactivation of Herpes zoster (HZ; shingles) were iron- and vitamin D deficiency and that HZ reactivation was prevented by supplementation with iron and vitamin D in maintenance hemodialysis patients (Chao et al. 2012). **Vitamin B12/folate methylation metabolism **and production of S-adenosyl methionine is critical for EBV latency through epigenetic methylation of the viral genome (Guo et al. 2022). Methylation metabolism is an obligatory requirement for energy production in mitochondria (van Rensburg et al. 2021a) and for myelin production by oligodendrocytes, since myelin basic protein has to be methylated (Kim et al. 1997). **Oxidation and Antioxidants:** Induction of reactive oxidative species (ROS) is associated with the lytic EBV cycle (Lassoued et al. 2010; Sausen et al. 2021). Multiple antioxidative nutrients inhibit EBV reactivation: Vitamin C, Vitamin D, Resveratrol, Luteolin, Apigenin Astragalus extract, Epigallocatechin-3-gallate, L-arginine, Sulforaphane, Baicalein, Rutamarin, Quercitin etc. (Pennisi et al. 2024; Kerr 2019; Wu et al. 2013. Wu et al. 2022). MS disability prevention is significantly associated with the amount of fruits and vegetables ingested daily (Davis et al. 2014). **Hypoxia** (low cellular oxygen) activates HIF-α, which regulates the life cycle of EBV and promotes lytic antigens (Kraus et al. 2017). HIF-1α directly binds to the promoter of the EBV primary latent-lytic switch BZLF1 gene, Zp (Kraus et al. 2017). Hypoxia is a risk factor for MS due to inadequate provision to brain cells of oxygen for energy production in mitochondria, inducing death of oligodendrocytes. White matter lesions occur primarily in brain regions where the blood vessels are thin and fragile (watershed regions) (Martinez Sosa and Smith 2017). Oxygen provision to the brain is enhanced through exercise (Kemp et al. 2023) and ozone therapy (Martinez Sosa and Smith 2017). **Lack of exercise and EBV reactivation** In male adolescents, strenuous sports were associated with a decrease in EBV antibody levels (p=0.012), while an additional hour per week spent viewing videos was associated with an increase in EBV antibody levels (p=0.026) (Lee 2016). In females, individual sports were also significantly associated with decreased antibodies (Lee 2016). Lack of exercise and a sedentary lifestyle in pwMS is significantly associated with decreased blood flow and increased intima media thickness (IMT) (Kemp et al. 2023). Increased IMT in pwMS is highly significantly associated with elevated disability (EDSS) in MS (Kemp et al. 2023). Increased blood flow through exercise leads to remission in pwMS (Kemp et al. 2023; Johannes et al. 2023; Jaftha et al. 2024). **Smoking** Smoking repeatedly reactivates EBV (Hu et al. 2019). Compared to non-smokers, smokers have a higher EBV viral load and higher levels of anti-EBV antibodies, indicating viral reactivation (Hedström et al. 2020a) **Allergies, environmental toxins and infections** Allergies and environmental toxins activate EBV (Olson et al. 1986; Hedström et al. 2023). Apart from viral infections such as Human simplex virus-1 (HSV), HIV and SARS-CoV-2, bacterial infections such as Aggregatibacter (which causes periodontal infections) and Helicobacter pylori are also associated with EBV reactivation (Indari et al. 2024). VZV and HHV-6 are risk factors for MS (Khalesi et al. 2023). EBV reactivation was found in acute COVID-19 infection as well as patients with long COVID-19 symptoms (Sausen et al. 2021) **Food sensitivity ** Some pwMS have sensitivities to gluten, lactose or lectins and experience improvements of MS symptoms such as fatigue if they avoid these in their food (Wahls et al. 2021). EBV gp42 glycoprotein is a member of the C-type lectin superfamily of proteins (Mullen et al. 2002). Published diets for MS emphasize a high intake of nutrient- and fiber-rich foods and plant-derived phytochemicals that are known to be beneficial to the gut microbiota that modulate neuroinflammation (Wahls et al. 2021; Katz Sand et al. 2023; Mirza et al. 2024). **Fatty acids and Lipid Profiles** Some short-chain fatty acids cause reactivation of EBV, while several medium-chain fatty acids inhibit lytic activation of EBV *in vitro* (Gorres et al. 2014). Saturated fat intake is associated with greater incidence and prevalence of MS as well as greater disability (Swank and Grimsgaard 1988). Saturated fat intake is also associated with obesity (Davis et al. 2014), which is in turn associated with infectious mononucleosis (Hedström et al. 2015), EBV antibodies and MS risk (Hedström et al. 2020b; Hedström 2023). Lipid profiles (cholesterol, HDL, LDL) as well as oxidized LDL are associated with adverse clinical and MRI outcomes in MS (Weinstock-Guttman et al. 2013; Zhornitsky et al. 2016). **Unsaturated fatty acids** possess potent immunomodulatory effects for preventing inflammation and production of inflammatory cytokines that could modulate the EBV latent-lytic cycle and prevent disability in MS (Hedström 2023). Evening primrose oil supplementation prevented disease worsening in pwMS who had the *FABP2* (rs1799883; 2445G>A, A54T) variant (Johannes et al. 2023). In pwMS, omega-3 and fish oils supplementation reduced the relapse rate, inflammatory markers, and improved quality of life (AlAmmar et al. 2021). Fish consumption and omega-3 supplementation were associated with improved quality of life and less disability while flaxseed oil supplementation was associated with over 60% lower relapse rate (Jelinek et al. 2013). The concentration of arachidonic acid (an n-6 PUFA) in erythrocyte membranes of pwMS was significantly inversely associated (p < 0.001) with the EDSS in pwMS (Hon et al. 2009), while increased plasma levels of n-6 PUFAs significantly decreased susceptibility to MS (Dunlop et al. 2024). The risk for disability progression in pwMS is exacerbated by an increased ratio of saturated versus unsaturated dietary fat intake (Swank and Grimsgaard 1988). PUFA deficiency in pwMS is mitigated by ingesting both n-3 and n-6 supplements as part of a structured dietary and supplementation program showing improved disability scores in pwMS (van Rensburg et al. 2021b; Johannes et al. 2023). **Stress** Psychological distress (Yamanashi et al. 2022) as well as perceived stress and pain are associated with EBV reactivation (Sausen et al.^2021^). The cellular immune response is impaired during stressful situations such as work stress (Uchakin et al. 2011), marital stress, student examination stress, anxiety or fear of abandonment and rejection, as well as loneliness, causing EBV reactivation (Kerr 2019). In contrast, increased perceived social support correlated with a decrease in the EBV antibody titer (Sausen et al. 2021). Stress is an aggravating risk factor for MS and MS relapses while controlling stress by regular exercise improves blood flow and alleviates stress and pain (Jaftha et al. 2024). **Inflammation** There is a concerted effort to investigate the underlying inflammatory mechanisms and develop new strategies for resolving smouldering inflammation in MS. Kocot et al. (2025) found that immunogenic cell death of oligodendrocytes in the vicinity of smouldering inflammation, characterized by activated microglia and macrophages, showed a signature of pyroptosis. Ma et al. (2025) showed that EBV infection was a driver of pyroptosis via the upregulation of glycolysis. Future research should focus on whether prevention of EBV reactivation could ameliorate smouldering inflammation, the primary driver of MS disability progression (Giovannoni et al. 2022).	

## Data Availability

No datasets were generated or analysed during the current study.

## Declarations

### Use of AI

No writing assistance or AI program was used to write this article. The manuscript was assessed by Turnitin to identify unintentional similarity to previously published work.
